# Characterization of protein arginine methyltransferase of TgPRMT5 in *Toxoplasma gondii*

**DOI:** 10.1186/s13071-019-3464-1

**Published:** 2019-05-08

**Authors:** Min Liu, Fen-Xiang Li, Chun-Yuan Li, Xiao-Cong Li, Long-Fei Chen, Kun Wu, Pei-Liang Yang, Zhi-Fa Lai, Ting-kai Liu, William J. Sullivan, Liwang Cui, Xiao-Guang Chen

**Affiliations:** 1 Department of Pathogen Biology, Guangdong Provincial Key Laboratory of Tropical Disease Research, School of Public Health, Southern Medical University, Guangzhou, 510515 Guangdong People’s Republic of China; 2 Laboratory Animal Research Center, Nanfang Hospital, Southern Medical University, Guangzhou, 510515 Guangdong People’s Republic of China; 3 Futian Center for disease control and prevention, Shenzhen, 518040 Guangdong People’s Republic of China; 4 Department of Pharmacology & Toxicology, Indiana University School of Medicine, Indianapolis, IN USA; 5 Department of Microbiology & Immunology, Indiana University School of Medicine, Indianapolis, IN USA; 6 Department of Entomology, Pennsylvania State University, 501 ASI Building, University Park, PA 16802 USA

**Keywords:** Histone, Chromatin, Parasites, Epigenetics, Methylation, Bradyzoites

## Abstract

**Background:**

Protein arginine methylation is a prevalent post-translational modification. The protein arginine methyltransferase family (PRMT) is involved in many cellular processes in eukaryotes, including transcriptional regulation, epigenetic regulation, RNA metabolism, and DNA damage repair. *Toxoplasma gondii*, an opportunistic protozoan parasite, encodes five conserved PRMTs. PRMT5 is thought to be responsible for substantial PRMT activity in *T. gondii*; however, it has not yet been characterized.

**Methods:**

We tagged the 3′ end of the endogenous TgPRMT5 genomic locus with sequence encoding a 3X hemagglutinin (HA) epitope. IFA and WB were performed to check the expression and subcellular localization of TgPRMT5 in tachyzoites and bradyzoites. *In vitro* methylation assays were performed to determine whether endogenous TgPRMT5 has arginine methyltransferase activity.

**Results:**

IFA and WB results showed that *T. gondii* PRMT5 (TgPRMT5) was localized in the cytoplasm in the tachyzoite stage; however, it shifts largely to the nuclear compartment in the bradyzoite stage. The *in vitro* methylation showed that TgPRMT5 has authentic type II PRMT activity and forms monomethylarginines and symmetric dimethylarginines.

**Conclusions:**

We determined the expression and cellular localization of TgPRMT5 in tachyzoites and bradyzoites and confirmed its type II PRMT activity. We demonstrated the major changes in expression and cellular localization of TgPRMT5 during the tachyzoite and bradyzoite stages in *T. gondii*. Our findings suggest that TgPRMT5 protein may be involved in tachyzoite-bradyzoite transformation.

## Background

*Toxoplasma gondii* (*T. gondii*) is an obligate intracellular parasite of the phylum Apicomplexa. This species is the causative agent of toxoplasmosis, which is estimated to infect 16–40% of the population of the USA and up to 80% of the population in other countries [1]. *Toxoplasma gondii* can differentiate from a rapidly replicating tachyzoite stage to a latent cyst form, the bradyzoite stage. Bradyzoite cysts can remain in tissues of the host for its lifetime without health consequences. However, when the host immunity is attenuated, bradyzoites can revert to actively replicating tachyzoites, causing life-long chronic infection. Thus, understanding the molecular mechanisms underpinning conversion between these stages may identify novel targets for clinical treatment.

Stage-specific gene expression is controlled by the concerted actions of specific transcription factors and repressors, translational repression, and epigenetic mechanisms [2]. Significant alterations in the expressed transcriptome and remodeling of chromatin structure, a major mechanism restricting and regulating access to genomic DNA, are occurring during the conversion between the tachyzoite and bradyzoite stages. *Toxoplasma gondii* has a full complement of histone-modifying enzymes, histones and variants [3]. Characterization of the *T. gondii* histone-modifying enzymes will provide a better understanding of the role of epigenetic regulation in gene expression in pathogenic apicomplexan parasites like *T. gondii*.

Gene expression can be negatively or positively modulated by methylation of arginine and lysine residues in histones. Moreover, both arginine and lysine methyltransferases are proposed to play an important role of gene expression regulators in *T. gondii* [4].

Structurally, each protein arginine methyltransferase family (PRMT) shares a conserved methyltransferase domain with subdomains for binding to S-adenosyl-L-methionine (SAM), a methyl donor and substrate proteins. Five putative arginine methyltransferases have been revealed by the bioinformatic analysis of the *Toxoplasma* genome sequence (http://www.toxodb.org) [5], and two PRMTs have been characterized in *T. gondii* [4–6]. PRMT1 (TGME49_219520) is reported to mediate methylation of arginine 3 of H4, while PRMT4 (TgCARM1) catalyzes methylation of arginine 17 of H3 and has been attributed to gene activation [5]. PRMT5 is a type II methyltransferase associated with transcriptional repression in other species [7, 8]. It is associated with multiple protein complexes and mediates diverse functions including RNA processing, transcriptional regulation, and muscle as well as germ line differentiation [9]. However, in *T. gondii,* the functions of PRMT5 remain poorly understood.

Here, we report the identification and characterization of the PRMT5 homolog, referred as TgPRMT5, in *T. gondii*. *TgPRMT5* (TGGT1_215560) is located on chromosome X, spans 2940 bp, and contains no introns. It encodes a protein of 979 amino acids with a predicted molecular mass of 107 kDa. The PROSITE database (http://us.expasy.org/prosite/) was used to search for predicted protein motifs in TgPRMT5. TgPRMT5 contains a conserved SAM-dependent methyltransferase PRMT-type domain as the PROSITE database showing it ranging from 601 to 957 amino acids. TgPRMT5 is an evolutionarily conserved protein with type II PRMT activity toward histone H3 arginine-26 (H3R26) and histone H4 arginine-3 (H4R3). Mammalian PRMT5 localizes to both the cytoplasm and the nucleus [10]. It has been proposed that PRMT5 relocates from nucleus to cytoplasm, where it may play a role in pluripotency regulation [11, 12]. TgPRMT5 is expressed in both tachyzoite and bradyzoite stages, and it is differentially localized between these stages, suggesting that TgPRMT5 might play a role in tachyzoite-bradyzoite conversion.

## Methods

### Parasite culture and nucleic extraction

The avirulent Pru*∆ku80* (Prugniaud) strain of *T. gondii* was maintained by serial passage in human foreskin fibroblasts (HFF) cultivated in Dulbecco’s modified Eagle medium supplemented with 1% (v/v) heat-inactivated fetal bovine serum and 25 μg/l gentamicin antibiotic (Life Technologies). To induce bradyzoite formation, approximately 50,000 tachyzoites were inoculated onto confluent HFF monolayers in T25 flasks with culture medium. Two to three hours post-infection, the culture medium was replaced with a pH 8.2 medium, which was replaced daily. Parasite DNA was isolated from the parasite pellet by proteinase K digestion and phenol/chloroform extraction.

### Plasmid construction and parasite transfection

We modified the endogenous TgPRMT5 genomic locus to contain additional sequence at the 3′ end that added a 3X hemagglutinin (HA) epitope onto the C-terminus of TgPRMT5. Pru*∆ku80* genomic DNA was used to amplify a 1.5 kb fragment at the PRMT5 3′ end using the primers PRMT5HA_F (5′-TAC TTC CAA TCC AAT TTA ATG CGT CTC TAC CAC TGC GTT TTC C-3′) and PRMT5HA_R (5′-TCC TCC ACT TCC AAT TTT AGC TTT TCC GAT GAA GTAA TGT TT-3′) containing ligation-independent cloning sequences (underlined). This PRMT5 fragment was inserted into the pLIC_HAx3_DHFRTs endogenous tagging vector such that the TgPRMT5 coding sequence was fused in frame with the epitope coding region. The pLIC_PRMT5_HAx3_DHFRTs construct was confirmed by sequencing. For transfection, 30 μg pLIC_PRMT5_HAx3_DHFRTs plasmid was linearized by overnight digestion with *SgrA*I within the PRMT5 homologous region followed by ethanol precipitation. Pru*∆ku80* tachyzoites were transformed with the linearized construct by electroporation. Subsequent to overnight growth in the HFF, parasite cultures were treated with 1.0 μM pyrimethamine [13]. Drug-resistant parasites were cloned by limiting dilution and screened using western blot and immunofluorescence for expression of HA-tagged TgPRMT5.

### Protein expression and purification

Full-length cDNAs encoding TgH3 (TGGT1_261240) and TgH4 (TGGT1_239260) were amplified by polymerase chain reaction using the primer pairs TgH3_F/TgH3_R (5′-AAA GAA TTC ATG GCG CGC ACC AAG -3′)/(5′-TCC GAG CTC TTA AGA CCG TTC ACC AC-3′) and TgH4_F/TgH4_R (5′-AAA GAA TTC ATG TCG GGC CGA GGC AAG-3′)/(5′-TCC GAG CTC TTA ACC ACC GAA ACC GTA G-3′) and cloned at the *EcoR*I and *Sac*I sites of vector PET-32a (Novagen, Madison, WI, USA) according to the manufacturer’s instructions to produce His-TgH3 and His-TgH4. Recombinant proteins were expressed in E.coli strain BL21 (DE3). Induction was performed by adding 0.1 mM IPTG for 4 h at 37 °C. Recombinant proteins were purified from 1 l of culture using Ni-NTA Fast Start Kit (Sigma-Aldrich, Taufkirchen, Germany). Purified recombinant TgH3 and TgH4 (rTgH3 and rTgH4) proteins were used for the *in vitro* methylation assay.

### Western blot

Tachyzoites were purified from infected HFF monolayers by scraping and passage through an 18G needle 10 times. Bradyzoites were purified from host cell debris by filtration through a 25 mm Nuclepore Track-Etched Polycarbonate Membrane circle with a 3.0 µm pore size (GE Healthcare, Madison, WI, USA) into a conical tube. Parasites were then pelleted by centrifugation and washed with cold PBS at 4 °C. Antibody to *Toxoplasma* BAG1 (1:1000) was used to detect protein-specific expression in the bradyzoites. *Toxoplasma* β-tubulin expression detected by specific polyclonal antibody (1:1000) served as a protein loading control. The antibody to BAG1 and anti-β-tubulin antibody were provided by Dr David Sibley (Washington University, St. Louis, MO, USA) and Dr Louis Weiss (Albert Einstein College of Medicine, NY, USA) respectively. To estimate the distribution of TgPRMT5 in the cytoplasmic and nuclear compartments of parasites, subcellular fractionations were performed using the NE-PER^TM^ nuclear and cytoplasmic extraction kit (Pierce, Rockford, IL, USA) according to the manufacturerʼs instructions. Parasite lysates (20 μg) of tachyzoites or bradyzoites were separated by SDS/PAGE (10%) and transferred to nitrocellulose membranes. Western blots were carried out using rat anti-HA antibody (Roche, CA, USA) (1:2000) and rabbit anti-H4 antibody (Merck Millipore, Darmstadt, Germany) (1:1000) as primary antibodies and horseradish peroxidase-conjugated goat anti-rat IgG (1:3000) and anti-rabbit IgG (1:3000) as secondary antibodies. The results were visualized using the ECL detection system (GE Healthcare).

### Indirect immunofluorescent assay (IFA)

For IFA, the infected HFF monolayers grown on coverslips were fixed in 4% paraformaldehyde for 20 min at room temperature. They were then permeabilized for 10 min in PBS containing 3% BSA and 0.2% Triton X-100 and blocked for 1 h in PBS with 3% BSA. The infected HFF monolayers were first probed with rabbit anti-HA antibody (Sigma) (1:500) and anti-BAG1 antibody (1:100). Secondary antibodies were FITC-labeled anti-rabbit IgG (Sigma-Aldrich, Taufkirchen, Germany) and TRITC-labeled anti-mouse IgG (Sigma-Aldrich, Taufkirchen, Germany). Fluorescent images were obtained using a Nikon ECLIPSE E600 epifluorescence microscope.

### Immunoprecipitation

To isolate epitope-tagged proteins, immunoprecipitations were performed using parasite lysates. Typically, 300–500 μg lysate was used for each immunoprecipitation reaction. Rat anti-HA antibody (Roche) was used for coupling with the Pierce Crosslink IP Kit (Thermo Fisher Scientific, Rockford, IL, USA) according to the manufacturer’s instructions. Lysates of the TgPRMT5-HA strain were immunoprecipitated with anti-HA antibody. The immunoprecipitated complexes were then used for an *in vitro* methylation assay.

### *In vitro* methylation assay

*In vitro* methylation assays were performed at 30 °C for 2 h in 20 μl methylation-assay buffer (50 mM Tris-HCl, pH 8.5, 20 mM KCl, 10 mM MgCl_2_, 1 mM DTT, 250 mM sucrose and 1 mM PMSF) containing 5 μg free calf core histones (H2A, H2B, H3 and H4) or 2 μg purified thioredoxin-6 × His *T. gondii* core histones H3 (rTgH3) and H4 (rTgH4) as substrates and 2 μg purified HA-tagged TgPRMT5. Reactions were stopped by the addition of LDS sample buffer (4×) and boiling for 5 min. Proteins were resolved by SDS/PAGE (15% gel) and transferred onto PVDF membranes. Immunoblot assays were performed using the following rabbit polyclonal antibodies: anti-monomethyl H3R26 (ab130898; Abcam, CA, USA), anti-symmetric dimethyl H3R26 (ab127095; Abcam, CA, USA), anti-monomethyl H4R3 (ab17339; Abcam, CA, USA), and anti-symmetric dimethyl H4R3 (ab5823; Abcam, CA, USA) at 1 μg/ml concentration and horseradish peroxidase-conjugated goat anti-rabbit IgG (Boster Bio-Tech, Wuhan, China) (1:2000) as the secondary antibody. The results were visualized with the ECL detection system.

## Results

### Fusion of HA-tag to endogenous TgPRMT5

**Fig. 1:**
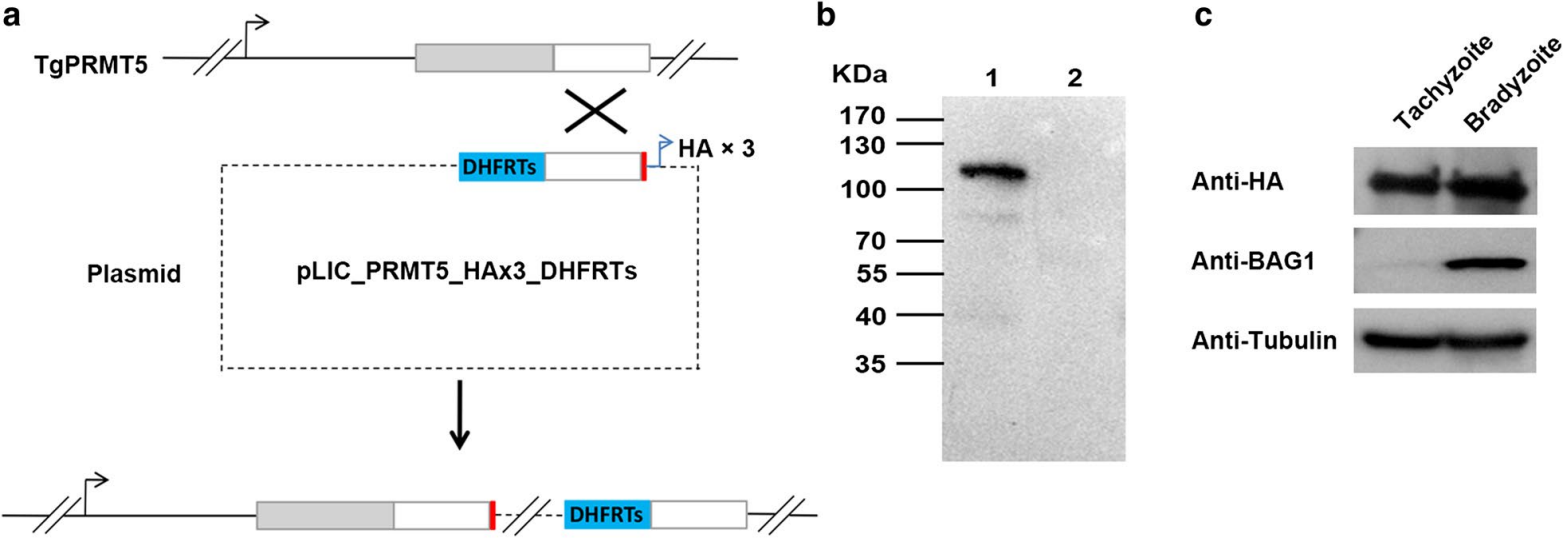
Confirmation of the C-terminal HA-tagging of the endogenous TgPRMT5 locus in the Pru strain. **a** Predicted integration event of HA fusion at the endogenous TgPRMT5 locus. Top: TgPRMT5 locus on chromosome X. Grey boxes represent the front part of TgPRMT5 gene; white boxes represent the TgPRMT5 region used for homologous recombination in the transfection plasmid; red boxes indicate HAx3-tagged. Middle: the plasmid pLIC_PRMT5_HAx3_DHFRTs. Bottom: The resultant single-crossover event at the TgPRMT5 locus with integration of one copy of the plasmid. **b** Western blot with anti-HA antibody that bind to the HA-tag. The band of 110 kDa was detected in the HA-tagged TgPRMT5 clone. Lane 1: clone of HA-tagged TgPRMT5; Lane 2: wild-type Pru strain as negative control. **c** Expression of TgPRMT5 in tachyzoites and bradyzoites. The two stages of the parasite were differentiated by antibody against BAG1, a protein expressed specifically in the bradyzoite stage. Anti-β-tubulin antibody served as a protein loading control

To study the expression and subcellular localization of TgPRMT5 in tachyzoites and bradyzoites, we tagged the 3′ end of the endogenous genomic locus with sequence encoding a 3xHA tag in Pru*∆ku80* parasites (Fig. 1a). Successful tagging of the endogenous TgPRMT5 was confirmed by IFA and western blot. Western blot using anti-HA antibody detected a specific protein band of 110 kDa, consistent with the predicted size of TgPRMT5 (Fig. 1b). By contrast, this protein band was not detected in the control wild-type Pru strain parasites. Western blot revealed that TgPRMT5 was expressed in both tachyzoites and bradyzoites with slight enrichment in the latter stage (Fig. 1c).

### Subcellular localization of TgPRMT5

**Fig. 2:**
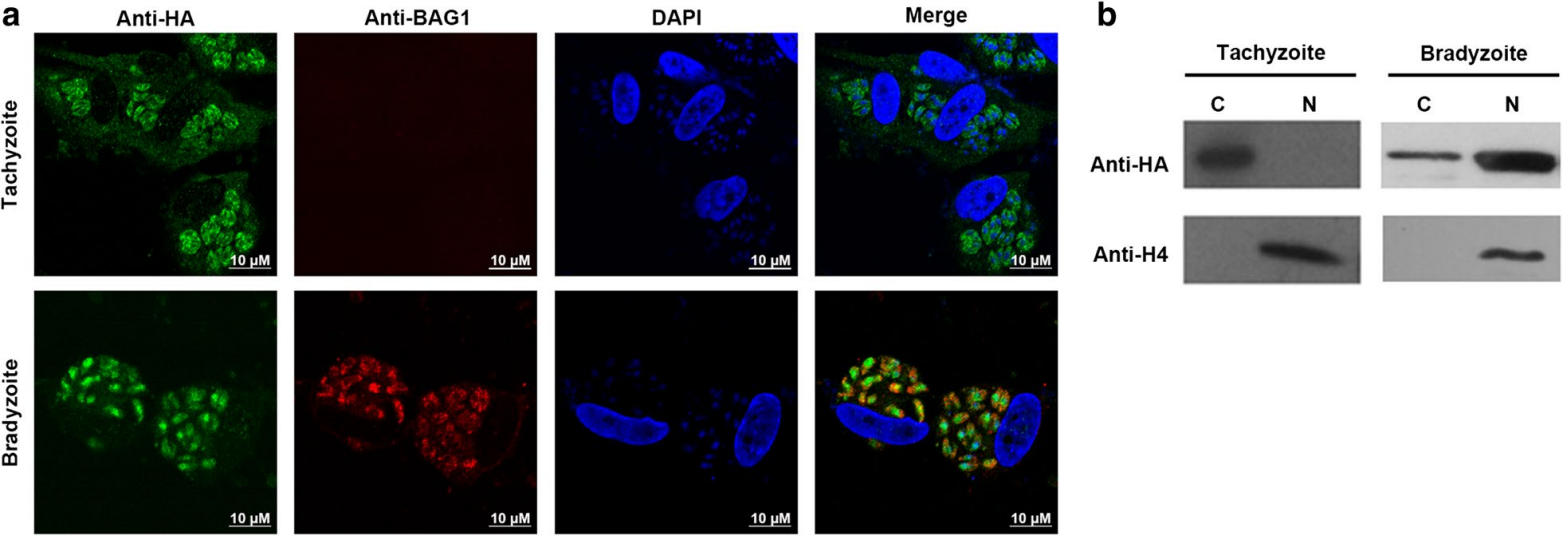
Subcellular localization of TgPRMT5 in tachyzoites and bradyzoites. **a** Localization of HA-TgPRMT5. Representative images of HA-TgPRMT5 in tachyzoites and bradyzoites. Nuclei were stained with DAPI. **b** Western blots of the parasite nuclear (N) and cytoplasmic (C) fractions, separated by 10% SDS-PAGE, and probed with anti-HA antibody (upper panel), and anti-H4 antibody (lower panel). *Scale-bars*: 10 µm

IFA was performed to check subcellular localization of TgPRMT5, and the TgBAG1 was specifically expressed in the cytoplasm in the bradyzoite stage as a bradyzoite marker. IFA results showed that TgPRMT5 was localized in the cytoplasm in the tachyzoite stage; however, it was mainly localized in the nucleus in the bradyzoite stage (Fig. 2a). Further analysis of the nuclear-cytoplasmic distribution of TgPRMT5 was performed using fractionated parasite lysates (Fig. 2b). Parasites were separated into nuclear and cytoplasmic fractions, and proteins from each fraction were resolved by SDS/PAGE and subjected to immunoblotting. The fidelity of the fractionation was confirmed by the predominant detection of histone H4 in the nuclear fraction (Fig. 2b). Consistent with the IFA observations, TgPRMT5 shifted from cytosolic to nuclear localization during conversion to the bradyzoite stage.

### TgPRMT5 enzyme activity

**Fig. 3:**
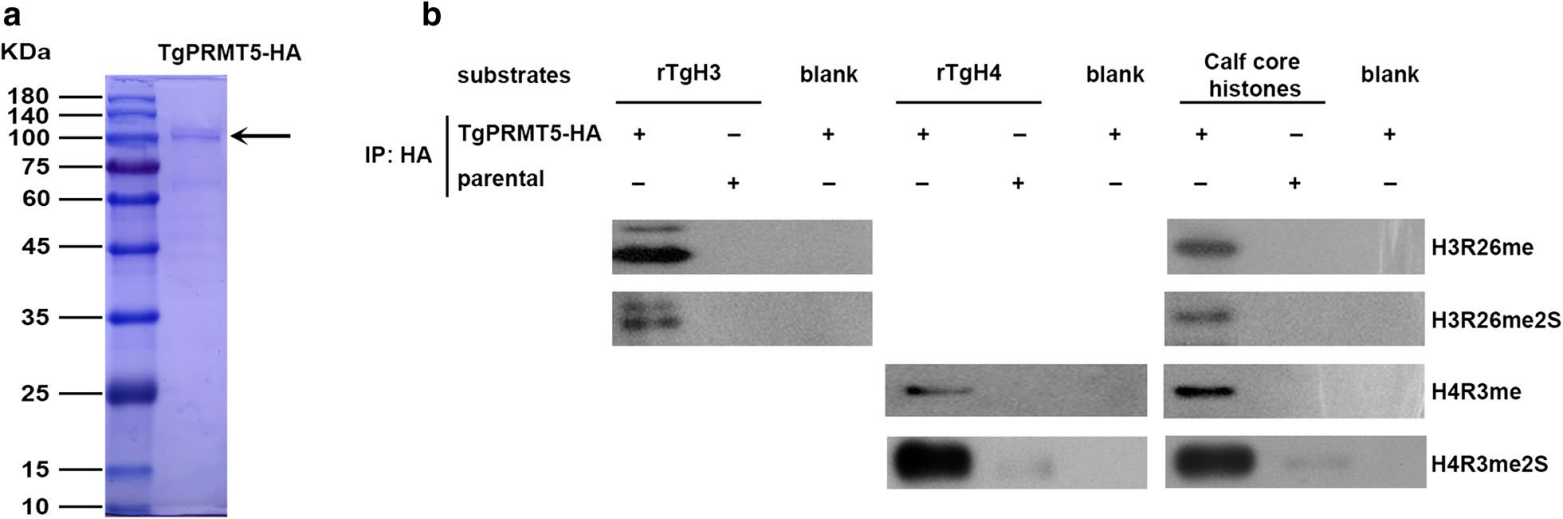
*In vitro* methylation of histones by purified HA-tagged endogenous TgPRMT5. **a** The HA-tagged endogenous TgPRMT5 were purified with an anti-HA antibody and checked in SDS/PAGE. **b** Immunoprecipitations using an anti-HA antibody were performed on parasite lysates made from TgPRMT5-HA and the parental Pru*∆ku80* line. The immunoprecipitated products were used *in vitro* methylation assays with *T. gondii* recombinant histones H3/H4 (rTgH3 and rTgH4) and calf core histones as substrates or without substrates (blank), and the reactions were separated by SDS/PAGE (15% gel). Immunoblots were performed using rabbit polyclonal antibodies as follows: anti-monomethyl H3R26, anti-symmetric dimethyl H3R26, anti-monomethyl H4R3 and anti-symmetric dimethyl H4R3

To determine whether endogenous TgPRMT5 has arginine methyltransferase activity, calf core histones H2A, H2B, H3 and H4 as well as purified *T. gondii* recombinant histones H3 and H4 (rTgH3 and rTgH4) were used as substrates in the methylation assay. Endogenous TgPRMT5-HA was purified using protein A/G beads with HA antibody. The purified products were checked in the SDS/PAGE gel and stained by Commassie Blue Staining Solution. The size of the main band was around 110 kDa, consistent with the predicted size of TgPRMT5-HA (Fig. 3a). Specific antibodies were used against monomethylated (MMA) and symmetrically dimethylated (SDMA) H3R26 or H4R3 to monitor TgPRMT5 activity by immunoblotting. Endogenous TgPRMT5-HA was shown to catalyze both H3R26 and H4R3 monomethylation and symmetric methylation, consistent with the prediction of this protein as a PRMT5 homolog (Fig. 3b).

## Discussion

Arginine methylation governs important cellular processes that impact growth, proliferation, differentiation, and development [9]. PRMT5 is the major type II methyltransferase and is widely distributed and highly conserved [14]. Unique among the PRMTs, PRMT5 works in concert with other cellular proteins including ATP-dependent chromatin remodelers and co-repressors to induce epigenetic silencing. Furthermore, PRMT5 has recently been implicated in control of growth-promoting and pro-survival pathways, thereby demonstrating its versatile enzymatic roles in epigenetic regulation of anti-cancer target genes and organelle biogenesis [9].

In the present study, we presented biochemical evidence demonstrating that TgPRMT5 has intrinsic type II PRMT activity, catalyzing the formation of MMA and SDMA. In the histone H4 N-terminus, TgPRMT5 can symmetrically methylate Arg^3^, a transcription repression marker [15]. These findings suggest that TgPRMT5 protein is a PRMT5 homolog and may participate in gene repression in *T. gondii.* TgPRMT5 was expressed in the tachyzoite and bradyzoite stages of the parasite, with the protein localization in cytoplasm in tachyzoites; however, it mainly localized in the nucleus in bradyzoites. In mammals, localization of PRMT5 differs between non-transformed and transformed cells. In most primary and immortalized cells, PRMT5 is primarily located in the cytosol with small amounts in the nucleus. In contrast, this distribution is reversed in transformed cells [16–18]. Therefore, PRMT5 localization and the substrates it targets in each cellular compartment appear to play a crucial role in the control of cell growth and proliferation. The differential subcellular localization of TgPRMT5 in tachyzoites and bradyzoites suggests that it may play a role in tachyzoite-bradyzoite conversion.

## Conclusions

In present study, we determined the expression and cellular localization of TgPRMT5 in tachyzoites and bradyzoites and confirmed its type II PRMT activity. We demonstrated the major changes in expression and cellular localization of TgPRMT5 during the tachyzoite and bradyzoite stages in *T. gondii*. These findings will provide a novel insight into addressing the mechanism of tachyzoite-bradyzoite transformation.

## Data Availability

Data supporting the conclusions of this article are included within the article.

## Abbreviations

PRU: Prugniaud
IFA: indirect immunofluorescent assay
PRMT: protein arginine methyltransferase
MMA: monomethylated
SDMA: symmetrically dimethylated
